# Effects of sacubitril/valsartan on cardiac reverse remodeling and cardiac resynchronization in patients with acute myocardial infarction

**DOI:** 10.3389/fcvm.2022.1059420

**Published:** 2023-01-13

**Authors:** Pei Yang, Xiaokang Li, Lijin Wang, Xinlei Wu, Chiyao Wang, Tian Li, Haiyan Wang

**Affiliations:** ^1^Department of Structural Heart Disease, The First Affiliated Hospital of Xi’an Jiaotong University, Xi’an, China; ^2^Jiajiang Integrated Warehouse, Leshan, Sichuan, China; ^3^Department of Cardiology, Tangdu Hospital, Fourth Military Medical University, Xi’an, China; ^4^School of Basic Medicine, Fourth Military Medical University, Xi’an, China

**Keywords:** sacubitril, valsartan, acute myocardial infarction, coronary intervention, retrospective study

## Abstract

**Introduction:**

In 2014, the PARADIGM-HF trial (Prospective Comparison of ARNI with ACEI to Determine Impact on Global Mortality and Morbidity in Heart Failure) has shown that sacubitril/valsartan can reduce the risk of hospitalization and death from cardiovascular causes more effectively than enalapril (an ACEI) in heart failure patients with reduced ejection fraction (HFrEF). Similarly, the PARADIGM-HF trial (Comparison of Sacubitril-Valsartan vs. Enalapril on Effect on NT-proBNP in Patients Stabilized from an Acute Heart Failure Episode) came to similar conclusions and extended the PARADIGM-HF trial results in 2019. Since then, numerous new studies have provided further insight in HFrEF, sacubitril/valsartan can reduce N-terminal pro-B-type natriuretic peptide (NT-proBNP) levels, increase left ventricular ejection fraction (LVEF), reverse ventricular remodeling, and reduce other non-fatal manifestations of clinical deterioration as compared to ACEI/ARB. However, few trials have compared the effects of these drugs in patients shortly after AMI. Therefore, it is necessary to further explore the clinical efficacy and safety of sacubitril/valsartan vs. valsartan in patients with AMI.

**Methods:**

We conducted an open-label, prospective, randomized controlled trial to determine the superiority in ameliorating ventricular remodeling and preventing of heart failure in patients with AMI after percutaneous coronary intervention (PCI), 148 patients were randomly assigned (85 to sacubitril/valsartan and 63 to valsartan).

**Results:**

LAV, LVDV, and LVSV were all decreased in the sacubitril/valsartan group when compared with before treatment, but there was no difference between the sacubitril/valsartan group and the valsartan group. In addition, compared with before treatment in the sacubitril/valsartan group, the heart global work index (GWI) and the global work efficiency (GWE) increased, while the heart global wasted work (GWW) decreased. Patients in the sacubitril/valsartan group have similar MACE and adverse side effects to those in the valsartan group.

**Conclusion:**

Sacubitril/valsartan has the same performance as valsartan in inhibiting ventricular remodeling and preventing heart failure after PCI in patients with AMI, and its clinical application is safe. It provides a clinical foundation for the application of sacubitril/valsartan in patients with AMI.

## 1. Introduction

The high risk of sudden death and high disability rate of acute myocardial infarction (AMI) posed a serious threat to people’s physical and mental health (1, 2). The main reason for this is coronary atherosclerosis, after which multiple triggers lead to the rupture of unstable plaques and thrombosis. Such as increased sympathetic excitability, enhanced body stress response, a high-fat diet, and emotional agitation. Fatal complications often occur in the early stage of AMI, such as acute heart failure, ventricular aneurysms, and cardiac rupture (3). Prompt initiation of vascular dredging in patients with AMI can save the dying myocardium and prevent the expansion of the infarct. In addition, administration of ACEI or ARB can prevent and reverse cardiac remodeling, reduce the risk of cardiovascular death or heart failure hospitalization, reduce the long-term risk of death, and improve the quality of life of patients (4–7).

Sacubitril/valsartan, as an angiotensin receptor-neprilysin inhibitor (ARNI), has been shown better effects than ACEI or ARB in many clinical trials among patients with chronic heart failure with reduced ejection fraction (8–12). However, whether there is a long-term benefit of sacubitril/valsartan in patients with acute myocardial infarction after PCI is still controversial (13–15). Several studies have shown that early application of sacubitril/valsartan is effective and safe in inhibiting ventricular remodeling in animal models of myocardial infarction (16–20). Therefore, it is also worthy of investigation and exploration to determine whether this efficacy and safety can continue to be prominent in clinical practice. Consequently, we conducted an open-label, prospective, randomized controlled trial to investigate the effects of sacubitril/valsartan in patients with AMI after PCI.

## 2. Methods

### 2.1. Study design and oversight

A total of 175 patients with AMI in the Second Affiliated Hospital of Air Force Military Medical University were recruited between November 2019 and February 2021, and finally, 148 patients were randomly assigned (85 to sacubitril/valsartan and 63 to valsartan). Follow-up and data analysis were carried out 1, 3, and 6 months after PCI. The design of this trial has been approved by the Ethics Committee of the Second Affiliated Hospital of Air Force Military Medical University (No. 201909-06) and registered in the China Clinical Trials Registration Center (ChiCTR2000041383).

#### 2.1.1. Inclusion criteria

(1) Individuals diagnosed with AMI, Killip I∼III grade. (2) Individuals between the ages of 20 and 80 years. (3) Individuals willing to provide written informed consent and who can comply with study procedures and follow-up.

#### 2.1.2. Exclusion criteria

(1) Individuals diagnosed with AMI, Killip IV grade, not eligible for PCI. (2) Individuals with severe hepatic insufficiency (Child-Pugh C grade) or severe renal insufficiency [eGFR < 30ml/(min⋅1.73m^2^)]. (3) Individuals with symptomatic hypotension (systolic blood pressure ≤ 90 mmHg). (4) Pregnant and lactating individuals. (5) Individual cognitive impairment. (6) Individuals have other serious illness or life expectancy < 6 months (21).

### 2.2. Therapeutic regimen

After the patients were admitted to the hospital, the basic information and risk factors of coronary heart disease were recorded through the interrogation. The type of myocardial infarction was determined according to the electrocardiogram (ECG), ultrasound, cardiac function, and biochemical examination. Coronary artery lesions were illustrated by the post-PCI records (the operation was performed by the attending physician or assistant director physician).

In addition to basic therapy, eligible patients were randomly assigned to receive treatment, in a double-blind manner, with either sacubitril/valsartan (100 mg twice daily) or valsartan (80 mg once a day). It needs to stop the drug for at least 36 h to avoid the occurrence of angioedema if they have taken ACEI/ARB drugs in the past. Based on the clinical manifestations of the patient and the occurrence of adverse reactions, it is necessary to determine whether to adjust the dose of the drug.

### 2.3. Evaluation

The primary outcome was the change from baseline to 6 months in LVEF or ultrasound indicators of cardiac resynchronization. Secondary outcomes, measured as the change from baseline to 6 months, were NT-proBNP, 6-minute walk distance (6MWD), systolic blood pressure (SBP), and diastolic blood pressure (DBP) (15). Safety parameters were assessed during the whole study based on MACE and adverse side effects. MACE included deaths from coronary heart disease, myocardial infarction, heart failure, severe arrhythmia, and recurrent angina pectoris. Adverse side effects included symptomatic hypotension (symptoms of hypotension with SBP ≤ 90 mmHg), angioedema, deterioration of renal function (serum creatinine ≥ 221 umol/L), and hyperkalemia (K^+^ ≥ 5.5 mmol/L). Trial visits were scheduled at 1, 3, and 6 months. During each visit, patients underwent comprehensive examinations, clinical safety assessments, and medication compliance analysis.

### 2.4. Statistical analysis

All statistical analyses were performed using the IBM SPSS 25.0 statistical software (IBM Inc., Chicago, IL, USA). After the Kolmogorov–Smirnov normality test, the measurement data that conformed to the normal distribution were expressed as the mean ± standard deviation. The comparison between the two groups was performed using the independent sample *t*-test. The comparison between before and after treatment was performed using the paired sample *t*-test. The measurement data that did not conform to normal distribution were expressed as quartile spacing. The Mann–Whitney *U* test was used for comparison between two groups, and the Wilcoxon signed-rank test was used for comparison before and after treatment. The count data were expressed as numbers and analyzed by Pearson’s Chi-squared test. Fisher’s exact test was used if the expected value was less than 5. Multiple linear regression models were used to study the effects of multiple variables. *P*-value < 0.05 was considered statistically significant (22).

## 3. Results

### 3.1. Baseline characteristics

**FIGURE 1:**
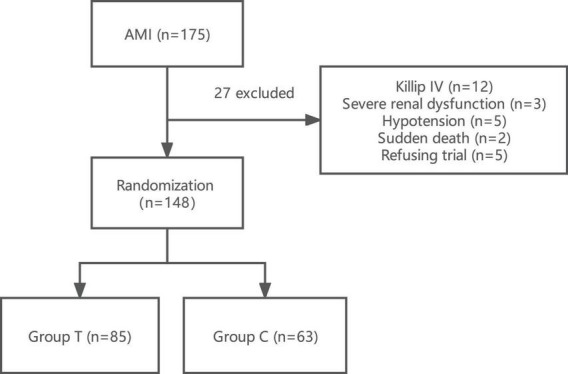
Flow chart of the research scheme showing the design of the study and detailed study selection process. Group T, sacubitril/valsartan group; Group C, valsartan group; AMI, acute myocardial infarction.

A total of 175 subjects were initially recruited. Among these patients, we excluded 12 patients with severe heart failure of Killip IV grade, 3 patients with severe renal insufficiency, 5 patients with symptomatic hypotension, 2 patients with sudden postoperative death, and 5 patients who refused to sign informed consent were excluded. Eventually, a total of 148 patients were included in the trial (Figure 1). These samples were randomly divided into the sacubitril/valsartan group (T group) and the valsartan group (C group).

The baseline characteristics of the patients were shown in Table 1. After random assignment, two groups were well-balanced in terms of demographic and clinical characteristics.

**TABLE 1:** The baseline characteristics of the patients.

Variables	T group (*n* = 85)	C group (*n* = 63)
Age (years)	59.07 ± 11.532	59.92 ± 12.019
Male	75 (88.2)	57 (90.5)
BMI (kg/m2)	22.96 ± 2.195	23.00 ± 2.540
Hypertension	44 (51.8)	36 (57.1)
Diabetes	18 (21.2)	17 (27)
Dyslipidaemia	16 (18.8)	10 (15.9)
Smoking	46 (54.1)	32 (50.8)
Family history	6 (7.1)	6 (5.1)
Myocardial infarction	7 (8.2)	3 (4.8)
Ischemic stroke	7 (8.2)	7 (11.1)
Valvular disease	1 (1.2)	1 (0.9)
Atrial fibrillation	3 (3.5)	1 (1.6)
STEMI	51 (60.0)	39 (61.9)
**Culprit vessel**		
LAD	56 (65.9)	39 (61.9)
LCX	4 (4.7)	5 (7.9)
RCA	25 (29.4)	19 (30.2)
**Killip classification**		
I	67 (78.8)	54 (85.7)
II	13 (15.3)	7 (11.1)
III	5 (5.9)	2 (3.2)
**Lesion artery number**		
Single-vessel	39 (45.9)	26 (41.3)
Double-vessel	29 (34.1)	20 (31.7)
Triple-vessel	17 (20.0)	17 (27.0)
CTO	6 (7.1)	3 (4.8)
**Laboratory analysis**		
CK-MB (ng/mL)	21.60 (3.38, 97.0)	27.10 (4.07, 94.2)
cTnI (ng/ml)	4.21 (1.04, 27.2)	3.04 (0.58, 20.7)
NT-proBNP (pg/ml)	869 (378, 2130)	588 (202, 1364)
eGFR (ml/min/1.73m^2^)	94.80 ± 20.132	95.72 ± 15.510
Creatinine (μmol/L)	71.98 ± 22.750	70.02 ± 17.917
**Drugs at discharge**		
Aspirin	64 (75.3)	54 (85.7)
Clopidogrel	81 (95.3)	61 (96.8)
Ticagrelor	4 (4.7)	2 (3.2)
Statins	85 (100)	63 (100)
β receptor blocker	82 (96.5)	62 (98.4)
**Diuretics**		
Furosemide	10 (11.8)	6 (9.5)
Spironolactone	17 (20.0)	11 (17.5)
Both	9 (10.6)	5 (7.9)

BMI, body mass index; LAD, left anterior descending artery; LCX, left circumflex artery; RCA, right coronary artery; CTO, chronic total occlusion; STEMI, st-segment elevation myocardial infarction; CK-MB, creatine kinase myocardial band; cTnI, cardiac troponin I; NT-proBNP, N-terminal pro-brain natriuretic peptide; eGFR, estimate glomerular filtration rate.

### 3.2. Comparison of echocardiographic results

**FIGURE 2:**
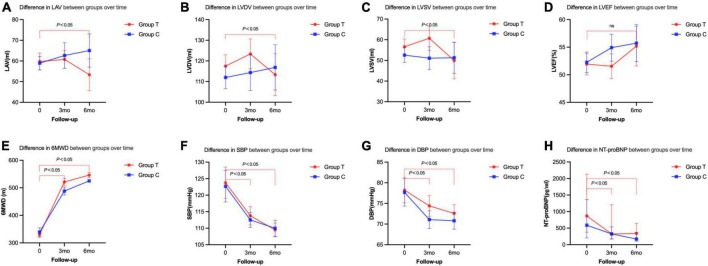
Primary and secondary end points changes between the two groups from the baseline to follow-up. **(A)** Difference in LAV between groups over time. **(B)** Difference in LVDV between groups over time. **(C)** Difference in LVSV between groups over time. **(D)** Difference in LVEF between groups over time. **(E)** Difference in 6MWD between groups over time. **(F)** Difference in SBP between groups over time. **(G)** Difference in DBP between groups over time. **(H)** Difference in NT-proBNP between groups over time.

All patients took the heart echocardiographic test and valued the left atrium volume (LAV), left ventricular end-diastolic volume (LVDV), and left ventricular end-systolic volume (LVSV) before and after the treatment. We found that LAV, LVDV, and LVSV in T group were significantly decreased (LAV, baseline 59.73 ± 19.01, 6 m 53.33 ± 16.94, *P* < 0.001; LVDV, baseline 117.47 ± 25.43, 6 m 113.33 ± 22.32, *P* < 0.001; LVSV, baseline 56.60 ± 17.49, 6 m 49.90 ± 19.36, *P* < 0.001) at 6 months after the treatment (Table 2 and Figure 2). There was no significant difference between the T group and the C group (LAV, T group 53.33 ± 16.94, C group 65.04 ± 19.95, *P* = 0.102; LVDV, T group 113.33 ± 22.32, C group 116.85 ± 27.17, *P* = 0.875; LVSV, T group 49.90 ± 19.36, C group 51.27 ± 18.70, *P* = 0.645) (Table 2).

**TABLE 2:** Primary and secondary end points changes between the two groups from the baseline to follow-up.

Variables	Baseline			Follow-up (3 m)				Follow-up (6 m)			
	T group (*n* = 85)	C group (*n* = 63)	*P-*value	T group (*n* = 71)	C group (*n* = 47)	*P*-value	*P*-value*	T group (*n* = 21)	C group (*n* = 26)	*P-*value	P-value*
LAV	59.73 ± 19.01	58.95 ± 12.94	0.780	60.76 ± 18.21	62.64 ± 21.06	0.557	0.357	53.33 ± 16.94	65.04 ± 19.95	0.102	<0.001
LVDV	117.47 ± 25.43	111.98 ± 21.60	0.169	123.39 ± 30.62	114.36 ± 29.48	0.682	0.154	113.33 ± 22.32	116.85 ± 27.17	0.875	<0.001
LVSV	56.60 ± 17.49	52.56 ± 13.98	0.120	60.70 ± 24.85	51.09 ± 18.86	0.240	0.201	49.90 ± 19.36	51.27 ± 18.70	0.645	<0.001
LVEF	51.93 ± 9.11	52.25 ± 7.38	0.817	51.55 ± 9.47	54.92 ± 8.36	0.147	0.963	55.19 ± 7.94	55.73 ± 8.33	0.480	0.230
6MWD	330 ± 49.4	338 ± 63.2	0.323	521 ± 63.7	487 ± 54.3	0.001	<0.001	546 ± 44.4	525 ± 15.2	0.012	<0.001
SBP	123.76 ± 22.06	122.70 ± 18.86	0.758	113.78 ± 12.89	112.52 ± 9.06	0.950	<0.001	109.49 ± 9.80	109.98 ± 9.60*	0.626	<0.001
DBP	78.20 ± 14.08	77.68 ± 13.16	0.821	74.40 ± 11.60	71.08 ± 8.54	0.175	0.003	72.58 ± 9.77	70.81 ± 8.11	0.352	<0.001
NT-proBNP	869 (378, 2130)	588 (203, 1364)	0.146	324 (158, 1210)	327 (195, 539)	0.335	0.012	338 (166, 643)	169 (105, 369)	0.254	0.002

LAV, left atrium volume; LVDV, left ventricular end-diastolic volume; LVESV, left ventricular end-systolic volume; LVEF, left ventricular ejection fraction; 6MWD, 6-minute walk distance; SBP, systolic blood pressure; DBP, diastolic blood pressure.

*P*-value: difference between groups at a single time point; *P*-value*: difference within T group over time.

### 3.3. Comparison of vital signs biochemical index

Compared with that before treatment, the index of 6MWD, SBP, DBP, NT-proBNP improved in both T and C group after 3 months and 6 months of treatment (*P* < 0.05). When we compared the T and C groups, SBP, DBP, and NT-proBNP did not show any significant differences. However, the 6MWD was significantly higher in T group than that in C group (*P* < 0.05) (Table 2 and Figure 2).

### 3.4. Cardiac resynchronization parameters

**FIGURE 3:**
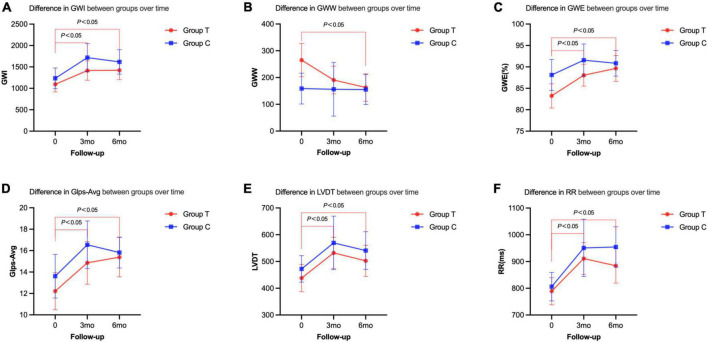
Cardiac resynchronization parameters of the two groups before and after the treatment at 3 or 6 months follow-up. **(A)** Difference in GWI between groups over time. **(B)** Difference in GWW between groups over time. **(C)** Difference in GWE between groups over time. **(D)** Difference in Glps-Avg between groups over time. **(E)** Difference in LVDT between groups over time. **(F)** Difference in RR between groups over time.

Compared with before treatment, the indexes of cardiac work (GWI, GWW, and GWE) in the sacubitril/valsartan group 6 months after the treatment were significantly improved (GWI: baseline, 1094.28 ± 428.53; 6 m, 1421.00 ± 421.23, *P* = 0.011; GWW: baseline, 265.16 ± 151.18; 6 m, 162.77 ± 99.99, *P* = 0.005; GWE: baseline, 83.24 ± 6.89; 6 m, 89.65 ± 5.85; *P* = 0.002). The left ventricular diastolic and systolic functions (GLS-AVG, LVDT, and VTIMV) were also significantly improved (*P* < 0.05) (Table 3 and Figure 3). However, there was no statistical significance on these indexes between the two groups (*P* > 0.05) (Table 3).

**TABLE 3:** Tissue synchronization imaging changes between the two groups from the baseline to follow-up.

Variables	Baseline			Follow-up (3 m)				Follow-up (6 m)			
	T group (*n* = 25)	C group (*n* = 19)	*P*-value	T group (*n* = 18)	C group (*n* = 7)	*P*-value	*P*-value*	T group (*n* = 17)	C group (*n* = 13)	*P*-value	*P*-value*
GWI	1094.28 ± 428.53	1234.21 ± 503.38	0.325	1415.11 ± 453.97	1717.29 ± 354.26	0.132	0.001	1421.00 ± 421.23	1617.39 ± 471.17	0.268	0.011
GCW	1491.80 ± 476.32	1487.00 ± 487.49	0.974	1697.39 ± 562.23	2039.43 ± 415.10	0.056	0.136	1674.65 ± 434.06	1868.23 ± 458.92	0.091	0.165
GWW	265.16 ± 151.18	158.79 ± 119.83	0.016	190.72 ± 104.08	156.14 ± 108.31	0.489	0.099	162.77 ± 99.99	155.23 ± 92.70	0.112	0.005
GWE (%)	83.24 ± 6.89	88.11 ± 7.53	0.031	88.06 ± 5.13	91.57 ± 4.08	0.943	0.023	89.65 ± 5.85	90.85 ± 4.96	0.340	0.002
LASr	0.20 ± 0.08	0.262 ± 0.108	0.039	0.25 ± 0.09	0.26 ± 0.10	0.463	0.012	0.25 ± 0.09	0.26 ± 0.08	0.203	0.121
LAScd	−0.09 ± 0.07	−0.13 ± 0.06	0.092	−1.44 ± 5.63	−0.126 ± 0.05	0.541	0.322	−0.11 ± 0.06	−0.13 ± 0.05	0.537	0.141
LASct	−0.11 ± 0.06	−0.14 ± 0.07	0.129	−0.11 ± 0.09	−0.13 ± 0.06	0.989	0.930	−0.137 ± 0.049	−0.12 ± 0.06	0.136	0.307
SV	55.68 ± 16.61	48.90 ± 12.71	0.146	56.06 ± 11.18	51.429 ± 9.13	0.520	0.118	54.06 ± 10.77	53.15 ± 11.99	0.714	0.566
CO	5.65 ± 8.10	3.705 ± 0.670	0.303	5.28 ± 7.22	3.30 ± 0.47	0.672	0.219	5.85 ± 9.86	3.332 ± 0.82	0.995	0.922
GLS-AVG	12.21 ± 4.18	13.61 ± 4.22	0.280	14.86 ± 4.01	16.54 ± 2.41	0.186	0.001	15.39 ± 3.58	15.82 ± 2.39	0.595	< 0.001
SPI	0.36 ± 0.09	0.34 ± 0.12	0.604	0.35 ± 0.09	0.35 ± 0.05	0.844	0.643	0.3 ± 0.094	0.35 ± 0.09	0.795	0.183
PSD	71.69 ± 26.73	66.45 ± 23.23	0.500	66.48 ± 21.16	59.24 ± 10.83	0.532	0.791	63.48 ± 21.37	64.53 ± 16.73	0.589	0.271
LVDT	437.92 ± 123.06	472.32 ± 102.52	0.330	532.00 ± 117.92	569.57 ± 107.95	0.670	0.009	502.38 ± 109.30	540.92 ± 116.66	0.912	0.034
RR	789.16 ± 123.14	806.26 ± 110.07	0.636	911.06 ± 119.57	950.86 ± 115.85	0.789	0.003	884.25 ± 121.50	954.62 ± 124.26	0.250	0.030
LVDT/RR	54.36 ± 8.77	58.00 ± 6.94	0.144	57.78 ± 7.24	59.43 ± 4.72	0.335	0.069	56.25 ± 5.86	55.92 ± 6.01	0.077	0.072
VTIMV	21.46 ± 5.37	21.72 ± 4.45	0.869	23.11 ± 4.35	22.17 ± 5.02	0.653	0.287	24.91 ± 5.40	22.56 ± 2.97	0.174	0.028
IVMD	11.60 ± 6.86	14.00 ± 14.67	0.515	8.41 ± 6.91	10.57 ± 5.41	0.760	0.090	8.63 ± 6.25	11.85 ± 12.71	0.748	0.171
SLD	0.0 (−42.0, 21.0)	10.0 (−5.0, 52.5)	0.426	−5.0 (−49.5, 26.8)	0.0 (−47.5, 36.5)	0.739	0.407	−10.0 (−43.0, 52.0)	−10.0 (−21.0, 0.0)	0.834	0.365
SPWMD	10.0 (−21.0, 73.0)	0.0 (0.0, 21)	0.933	10.5 (2.5, 57.8)	10.0 (−21.0, 78.5)	0.563	0.371	10.0 (0.0, 84.0)	42.0 (11.0, 83.0)	0.246	0.453
BMD	84.32 ± 50.22	67.90 ± 45.34	0.269	74.67 ± 51.37	96.14 ± 33.11	0.213	0.279	84.59 ± 44.00	83.46 ± 42.83	0.596	0.239
BS	34.72 ± 21.19	26.58 ± 17.94	0.185	30.22 ± 22.29	39.57 ± 15.02	0.120	0.231	36.65 ± 18.05	33.00 ± 17.57	0.697	0.141
aSMD	111.28 ± 48.24	88.11 ± 42.35	0.104	98.78 ± 59.11	115.43 ± 42.29	0.108	0.188	117.12 ± 44.595	112.62 ± 50.00	0.459	0.223
aSS	37.56 ± 18.78	27.90 ± 14.45	0.070	34.50 ± 22.58	40.29 ± 17.05	0.026	0.313	39.12 ± 16.94	37.23 ± 17.22	0.400	0.204

GWI, global work index; GWW, global wasted work; GWE, global work efficiency; GLS-AVG, global mean longitudinal strain of left ventricle; LVDT, left ventricular diastolic filling time; RR, R-R interval; VTIMV, velocity time integral of mitral valve; GCW, global constructive work; LASr, left atrial reservoir longitudinal; LAScd, left atrial conduit longitudinal; LASct, left atria contraction longitudinal; SV, stroke volume; CO, cardiac output; SPI, spherical index; PSD, peak strain time dispersion; IVMD, interventricular mechanical delay; SLD, septal lateral delay; SPWMD, septum posterior wall mechanical delay; BMD, basal max delay; BS, basal stdev; aSMD, all seg max delay; aSS, all segments stdev.

*P*-value: difference between groups at a single time point; *P*-value*: difference within T group over time.

### 3.5. Multiple linear regression analyses between GWI and key factors

**FIGURE 4:**
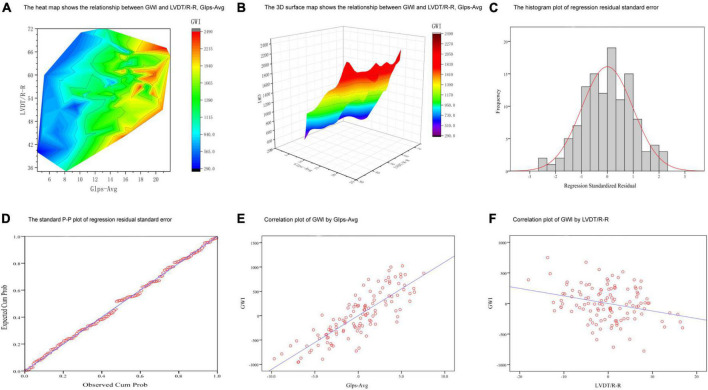
Multiple linear regression analyses between GWI and key factors. **(A,B)** The 3D graphs show the relationship between GWI and LVDT/R-R, GIps-Avg. **(C)** The histogram plot of regression residual standard error. **(D)** The standard P-P plot of regression residual standard error. **(E)** Correlation plot of GWI by GIps-Avg. **(F)** Correlation plot of GWI by LVDT/R-R.

A multiple linear regression model was used to screen the independent variables affecting GWI. GLS-AVG and LVDT/RR were identified as the main influencing factors (Supplementary Table 1). The levels of GLS-AVG and LVDT/RR were significantly associated with GWI (Figure 4). We also established equations for GWI with GLS-AVG and LVDT/RR to predict the improvement of the overall cardiac work (Supplementary Table 1).

### 3.6. MACE outcomes and adverse side effects

Hypotension was the most frequent adverse side effect (3.5% in the T group and 3.2% in the C group). And congestive HF was the most frequent MACE (2.4% in the T group and 4.8% in the C group). Comparing these two groups, there was no significant difference in the frequency of MACE and adverse side effects after the treatment (*P* > 0.05) (Table 4).

**TABLE 4:** Major adverse cardiovascular events and adverse side effects between the two groups.

Variables	Major adverse cardiovascular events			Variables	Adverse side effects		
	T group (*n* = 85)	C group (*n* = 63)	*P*-value		T group (*n* = 85)	C group (*n* = 63)	*P*-value
Cardiac death, n (%)	1 (1.2)	0.000	1.000	Hypotension, n (%)	3 (3.5)	2 (3.2)	1.000
Myocardial reinfarction, n (%)	1 (1.2)	2 (3.2)	0.575	Angioedema, n (%)	1 (1.2)	0.000	1.000
Congestive HF, n (%)	2 (2.4)	3 (4.8)	0.651	Renal cause, n (%)	1 (1.2)	1 (1.6)	1.000
Malignant arrhythmia, n (%)	0	0	1	Hyperkalemia, n (%)	1 (1.2)	1 (1.6)	1.000
Recurrent angina, n (%)	0	2 (3.2)	0.18	–	–	–	–
All, n (%)	4 (4.7)	7 (11.1)	0.249	All, n (%)	6 (7.1)	4 (6.3)	1.000

HF, heart failure.

## 4. Discussion

Cardiovascular diseases remain the main killer worldwide (23–25). Due to the high risk and high fatality rate of AMI, clinicians are constantly urged to explore how to diagnose, treat, and improve the prognosis as soon as possible to minimize the harm. We know that the total time of myocardial ischemia in patients with acute myocardial infarction determines the size and prognosis of myocardial infarction (26), and ventricular remodeling after myocardial infarction is also affected by a variety of risk factors (27). The risk of ventricular remodeling in patients with anterior wall myocardial infarction was 1.9 times higher than that of infarction at other sites (28). The risk of ventricular remodeling in patients with multi-vessel disease was 1.2 times higher than that in patients with single-vessel disease (29). The severity of chronic total occlusive disease (CTO) and valvular disease is closely related to the degree of cardiac remodeling (30, 31). In theory, early inhibition of ventricular remodeling could delay or prevent the progression of heart failure and reduce the risk of death and rehospitalization. According to the guidelines and consensus, all patients with acute myocardial infarction should use beta blockers or angiotensin converting enzyme inhibitors (ACEI)/angiotensin receptor antagonists (ARB) as early as possible if there is no contraindication (32, 33).

Based on the study of sacubitril/valsartan in patients with heart failure (34–36), most of the conclusions showed that it was effective in the treatment of hypertension (37), diabetes with chronic renal insufficiency, cardiac insufficiency caused by cardiotoxicity of chemotherapeutic drugs, and functional mitral regurgitation patients (38–42). The purpose of this study is to explore the role of sacubitril/valsartan in cardiac remodeling in patients with myocardial infarction.

This study found that the indexes of cardiac remodeling (LAV, LVDV, and LVSV) improved significantly in the sacubitril/valsartan group after 6 months of treatment, suggesting that sacubitril/valsartan has a significant effect on inhibiting myocardial remodeling in patients with myocardial infarction. This is consistent with the results of a recent meta-analysis confirming that sacubitril/valsartan is effective in improving cardiac remodeling (13, 43, 44). According to previous studies, valsartan was more effective in patients with anterior descending artery disease (45). With more than 60% of patients having anterior descending artery disease, our samples showed fairly high sensitivity to the treatment of valsartan. Our results demonstrated that there was no statistical difference in the indexes of cardiac remodeling between the two groups during the same period, suggesting that sacubitril/valsartan provided equal efficacy to valsartan.

B-type natriuretic peptide (BNP) and N-terminal B-type natriuretic peptide (NT-proBNP) in the family of natriuretic peptides are currently the most widely used biomarkers in the diagnosis and treatment of heart failure, whereas BNP and NT-proBNP detection results are affected by drugs. BNP is the substrate of the enkephalin enzyme, thereby when patients take sacubitril/valsartan, enkephalin inhibitor inhibits enzyme hydrolysis, increasing BNP concentration, while NT-proBNP is not affected by the enkephalin inhibitor. As an important indicator of the prognosis of patients with heart failure, NT-proBNP was also compared between and within groups. Compared with the baseline, the NT-proBNP of both groups decreased significantly after the treatment [T group, baseline 869 (378, 2130), 3 m 324 (158, 1210), 6 m 338 (166, 643), *P* < 0.05; C group, baseline 588 (203, 1364), 3 m 327 (195, 539), 6 m 169 (105, 369), *P* < 0.05]. However, there was no statistical difference between the two groups (3 m *P* = 0.335, 6 m *P* = 0.254). This result was not consistent with that of previous studies showing that there was a significant difference in NT-proBNP between sacubitril/valsartan and ACEI/ARB drugs at 4 weeks (46, 47). The reason for this result may be due to the fact that this study included patients with acute myocardial infarction, most of them did not have symptoms of severe heart failure with relatively low level of NT-proBNP and large inter-individual differences at the baseline.

Our study found that the GWI and the GWE of the patients after the treatment in the sacubitril/valsartan group increased significantly, while the GWW decreased significantly. It is suggested that sacubitril/valsartan increased the work efficiency of the heart by reducing the ineffective work, indicating that myocardial function is gradually recovering. Meanwhile, during the 6-month follow-up in the valsartan group, the GWI was significantly increased (baseline, 1234.21 ± 503.38; 6 m, 1617.39 ± 471.17; *P* = 0.001) and the GCW was also significantly increased (baseline, 1487.00 ± 487.49; 6 m, 1868.23 ± 458.92, *P* = 0.002). However, unlike the T group, the GWW was not significantly decreased (baseline, 158.79 ± 119.83; 6 m, 155.23 ± 92.70; *P* = 0.165) and the GWE of the heart (baseline, 88.11 ± 7.53; 6 m, 90.85 ± 4.96; *P* = 0.138) also showed no significant increase. Even though we did not get any significant difference on the indexes of heart work between the two groups after the treatment, the improvement of GWW and GWE in the T group without happening in the C group suggested that the treatment with sacubitril/valsartan could have better efficacy on the improvement of heart work. This could also explain the fact that patients in the T group got higher 6MWD with better cardiac work and increased activity tolerance.

During the study period, the incidences of malignant arrhythmia, renal insufficiency, and hyperkalemia in the test group were 0, 1.2%, and 1.2%, respectively, while those in the control group were 0, 1.6%, and 1.6%, respectively. There was no significant difference between these two groups. Compared with the control group, the incidence of symptomatic hypotension in the test group was higher (3.5%), and the incidence of myocardial infarction and heart failure was lower (1.2 and 1.4%, respectively), but these differences were not significant (*P* > 0.05). It may be due to the small sample size and relatively short follow-up time in this study. In addition, the use of β-blockers and spironolactone may also interfere with the efficacy of the trial group (22).

## 5. Conclusion

Overall, this manuscript bears cons. This small sample-size study was performed in a single center. With the extension of follow-up time, there bears loss to follow-up. Additionally, cardiac synchronous ultrasound examination is difficult to perform, and the data lack repetition.

Compared with valsartan, sacubitril/valsartan is also effective in inhibiting ventricular remodeling and preventing heart failure in patients with acute myocardial infarction after PCI, and its clinical application is safe. Our results provide a clinical basis for the application of sacubitril/valsartan in patients with acute myocardial infarction. The conclusion of this study still needs to include more patients for longer follow-up and further investigate the clinical efficacy and safety.

## Data availability statement

The original contributions presented in this study are included in this article/Supplementary material, further inquiries can be directed to the corresponding authors.

## Ethics statement

The studies involving human participants were reviewed and approved by Ethics Committee of the Second Affiliated Hospital of Air Force Military Medical University. The patients/participants provided their written informed consent to participate in this study.

## Author contributions

PY and XL performed the substantial contributions to conception, design, and drafted the manuscript. CW, TL, and HW revised the manuscript critically for important intellectual content and approved the final version to be published. All authors contributed to the article and approved the submitted version.
